# Efficacy and Cost-Benefit Analysis of Magnetic Resonance Imaging in the Follow-Up of Soft Tissue Sarcomas of the Extremities and Trunk

**DOI:** 10.1155/2021/5580431

**Published:** 2021-04-27

**Authors:** Daniel Kraus, Felix Oettinger, Jurij Kiefer, Holger Bannasch, G. Björn Stark, Filip Simunovic

**Affiliations:** ^1^Department of Plastic and Hand Surgery, Freiburg University Medical Center, Freiburg 79106, Germany; ^2^Department of Plastic, Hand und Aesthetic Surgery, Schwarzwald-Baar-Klinikum Donaueschingen, Donaueschingen 78166, Germany

## Abstract

There is no consensus regarding follow-up after soft tissue sarcoma (STS) treatment. This study examines the efficacy and the cost-benefit of MRI imaging for discovering recurrence. A retrospective analysis was performed, collecting data on patient demography, tumor characteristics, treatment, and follow-up. Imaging was correlated to the clinical course, and sensitivity, specificity, and predictive values were calculated. The number needed to screen and costs of finding recurrence are reported. Amongst 216 sarcomas, 73 (35%) exhibited local recurrence during a follow-up of 5.3 ± 3.5 years. 173 entities had complete MRI follow-up with 58 (34%) local recurrences. Thirty-three (57%) were discovered by MRI, 8 (14%) by clinical presentation, and 17 (29%) simultaneously. There was a sensitivity of 100.00%, a specificity of 89%, a positive predictive value of 32%, and a negative predictive value of 100% for detecting local recurrence with MRI. Our data confirm the modalities and intervals proposed by the German guidelines for sarcoma care. The recommended MRI intervals should not be extended. MRI is more cost-effective than clinical examination; still, both modalities should be performed together to discover the maximum number of recurrences.

## 1. Introduction

Soft tissue sarcomas are a heterogeneous group of rare malignant tumors of mesenchymal origin. They account for about 1% of malignancies in adults, with over 50 subtypes [1–3]. The European guideline of the European Society for Medical Oncology–European Reference Network (ESMO–EURACAN) and the German guideline on the clinical practice regarding diagnosis, treatment, and follow-up for soft tissue and visceral sarcomas describe that complete resection in combination with irradiation and, for rare chemosensitive tumors, chemotherapy, is the standard therapy [4–7]. There is consensus that sarcoma diagnosis and treatment should be conducted in specialized high-volume centers [1, 8, 9].

Despite adequate treatment, local recurrence occurs in 5 to 35% of patients [10, 11], which worsens the clinical outcome [12]. Since recurrences can be treated with repeated resection and radiotherapy, often enabling further limb-preservation and reducing disease-related mortality and morbidity, early detection of local recurrence is essential. There is, however, no agreement on the optimal surveillance protocol. A review of the international literature of 34 articles reported on 54 different follow-up strategies, ranging from clinical examination alone to whole-body positron emission tomography–computed tomography (PET-CT) [13]. 18F-Fluorodeoxyglucose positron emission tomography–magnetic resonance imaging (18F-FDG PET-MRI) combines the superior soft-tissue resolution of an MRI scan and the molecular sensitivity of an 18F-FDG PET. A recent review concludes that this promising modality has a higher accuracy for detecting local recurrence than MRI [14]. Our centre adheres to the German guideline on soft tissue sarcoma [5]. During the first two years, patients with high-grade sarcomas (≥G2) undergo quarterly magnetic resonance imaging (MRI) with gadolinium contrast enhancement and clinical examination, while patients with low-grade (G1) tumors undergo MRI and examination every 6 months. Within the next 3 years, the follow-up is conducted semiannually, continued with annual follow-ups for years 6–10.

An overwhelming majority of MRI scans reveal no pathological findings, and every follow-up is a considerable logistic and psychological burden for the patients [15]. In this study, we investigated the efficacy of MRI diagnostics in the follow-up of soft tissue sarcomas of the trunk and extremities. We hypothesized that the current standard of care results in an excessive number of MRI scans, which pose a significant strain on the patient and the health system. Sensitivity, specificity, and predictive values for MRI imaging were evaluated. The diagnostic quality of the MRIs performed internally at the university hospital, and those performed externally, was compared. Furthermore, the number needed to screen and the total healthcare-associated costs of MRI and clinical examination were calculated.

## 2. Materials and Methods

We conducted a retrospective analysis of patients treated at the Department of Plastic and Hand Surgery of the University Medical Center Freiburg. The inclusion criteria were patients diagnosed with a soft tissue sarcoma of the extremity and the trunk, who had completed at least a five-year follow-up. Data were compiled from medical records as well as radiology and pathology reports between 2003 and 2013. Patients undergoing the primary operation after 2013 were excluded because the follow-up was shorter than five years. Patients with intra-abdominal and retroperitoneal sarcomas were excluded. Patients with incomplete records, or those lost to follow-up, were excluded from the test accuracy calculation. The Ethics Committee of the University of Freiburg approved this study (282/19). The data that support the findings of this study are available on request from the corresponding author. The data are not publicly available due to privacy and ethical restrictions.

The data were collected anonymously. The sequence of treatment modalities was proposed by the interdisciplinary sarcoma board, and plastic surgeons specialized in sarcoma surgery performed the oncological resection and reconstruction. Our approach to treatment and surgical strategy was extensively published previously [1, 6, 8, 16–21]. This study analysed local recurrence and not distant metastases. The type of sarcoma was recorded, as well as anatomic location (proximal vs. distal extremity), the location of the tumor (superficial vs. deep), histological grading and size of the tumor, and the staging status of the patient. Details of treatment modalities were reported. The frequency of recurrence was examined and correlated to tumor location and grading. Radiology reports were classified into three categories: inconspicuous, unclear (cannot exclude recurrence), and suspected local recurrence. The clinical consequence of the latter two findings was documented and included: no consequence, shortening of the follow-up interval, surgical procedure, or other consequences (additional imaging study). If a biopsy was positive for recurrence, and it was followed by surgical resection, this sequence was recorded as one surgery, and the results of pathology on the end specimen were recorded. Positive tumor status was assigned to cases which had pathological diagnosis of recurrence. Negative tumor status was retrospectively assigned to MRI examinations that were followed by an unremarkable examination.

Four-field tables were used to determine the sensitivity, specificity, and predictive values of MRI scans for the diagnosis of sarcoma recurrence. Calculations were then performed separately for internal MRI diagnostics at the Freiburg University and external MRI examinations performed peripherally. The number needed to screen was calculated for the respective intervals of the MRI follow-up, i.e., the number of examinations needed to find a local recurrence [22]. To evaluate the costs of MRI diagnostics, the number needed to treat was multiplied by the costs of an MRI examination, which is €200. The quarterly cost for a clinical examination is €127.30. We corrected this to €124.58 since some examinations took place in the same quarter.

## 3. Results and Discussion

### 3.1. Patient Demography, Tumor Characteristics, and Factors Influencing the Occurrence of Local Recurrence

The postoperative course and the MRI follow-up of 211 patients with a total of 216 tumor entities were examined. 49% were female, and the average age at time of the first operation was 58.4 years. The most substantial proportion of the 216 sarcomas treated were liposarcoma (30%), followed by undifferentiated or unclassified sarcomas (26%), and fibroblastic or myofibroblastic sarcomas (21%). Smooth muscle sarcomas, skeletal muscle sarcomas, vascular tumors of soft tissue, nerve sheet sarcomas, and extraskeletal bone sarcomas were encountered in under 5% of the cases. Location of tumors, size, staging, *R* status, grading, and therapy modalities are reported in Table 1.

There was no difference in the frequency of recurrence concerning tumor location (proximal vs. distal: 22% and 23%, *p*=0.854). The likelihood of recurrence increased with tumor grading (16%, 23%, and 23% for G1, 2, and 3, respectively), but this result was also not significant (*p*=0.301 when comparing G1 and G3 tumors; *p*=0.287 when comparing G1 and G2/G3 tumors, Table 2).

### 3.2. Frequency and Time of Recurrence Onset

During the follow-up, 73 local recurrences were detected. For the period considered, this results in a mean of 0.34 local recurrences per tumor entity and an incidence of 0.064 local recurrences per tumor entity and year. 78% of the entities developed no local recurrence and 22% at least one local recurrence.

In contrast to the entire observation period, the period of MRI follow-up for the 173 entities was 5.17 years per tumor entity. The discrepancy between the total case number of 216 and the MRI follow-up case number of 173 is explained by the fact that some tumors did not require imaging for surveillance (cutaneous tumors) and that some cases had incomplete documentation. In this group, there was a total of 58 local recurrences (34%) and an incidence of 0.065 local recurrences per tumor entity and per year examined. 79% of the entities developed no local recurrence during the follow-up period, and 21% developed at least one recurrence.

**Figure 1:**
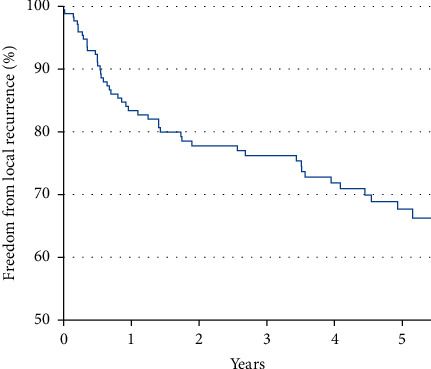
Survival without local recurrence after the initial operation, in years since surgery until the first local recurrence (event) or until death or end of follow-up (censoring).

Figure 1 depicts recurrence-free survival of patients without the development of local recurrence, depending on the time elapsed after the first operation. Patients who did not develop local recurrence were censored at the time of the last regular examination or death. After five years, 122 patients had either developed a local recurrence or had been censored from the graph, while 94 entities were further monitored. Thus, 36% of the patients developed at least one local recurrence within the first six years.

### 3.3. Discovery of Local Recurrence

**Figure 2:**
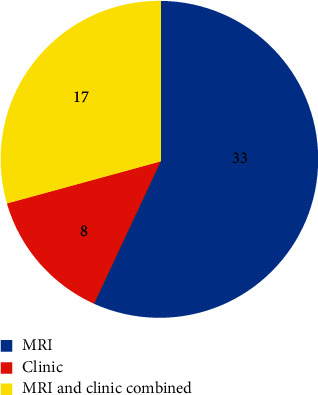
Modalities of discovering local recurrence in the period of the MRI follow-up.

Of the 58 local recurrences found within the MRI follow-up period, 57% (*n* = 33) were primarily detected by an MRI examination, 14% (*n* = 8) primarily by clinical examination, and 29% (*n* = 17) by both methods almost simultaneously (Figure 2). Clinical symptoms and findings included palpable swelling, pain, and restricted movement. All eight recurrences which were suspected in clinical examination could subsequently also be shown in the MRI examination. Clinical examination was more critical for the distal local relapses than for the proximal ones, even though this trend did not reach statistical significance (*p*=0.31). Clinical symptoms, such as pain, were also more common in distal local recurrences than in proximal ones. Furthermore, high-grade soft tissue sarcomas (G2 and 3) were more often noticed clinically or simultaneously by MRI and clinical examination, than G1 sarcoma (*p*=0.0428, Table 3).

A total of 1590 MRI examinations were carried out during the follow-up period. 89% of the MRI studies (*n* = 1416) were carried out internally at the University Medical Center Freiburg, whereas 11% (*n* = 174) were performed externally. The arithmetic mean of MRI examinations was 7.36 per entity across all 216 tumor entities. Follow-up was successfully completed in 51 entities (24%) after at least ten years of complete remission. 61 entities (28%) received follow-up for less than five years. Follow-up was not yet complete at the time of data collection for 46 entities (21%). For the other entities, the follow-up was aborted after five to ten years.

When the MRI report excluded recurrence, there was no change in the follow-up. When the radiologist suspected recurrence, there was no clinical consequence in 24% of the cases. Shortening of the follow-up interval was chosen in 29%, and a surgical procedure was performed in 42% of such situations. Uncertain results (inability to exclude recurrence) had no consequence in 66% of the cases, whereas in 31% the interval was shortened, and in 3% an operative procedure was carried out (Table 4).

90% of the MRI examinations (*n* = 1331) showed a negative tumor status. Of the remaining 9.70% of cases with local recurrence (*n* = 143), 68 MRI examinations were involved in finding newly occurring local recurrences and 75 observed previously known recurrences. The sensitivity of the striking findings is 85% and the specificity of the findings with exclusion of local recurrence is 89%. In this calculation, regarding sensitivity and specificity, the uncertain findings (second values in square brackets) do not flow into the numerator, but nevertheless into the denominator, since they do not make any clear statements regarding the tumor status, but form part of the population of examinations with positive or negative tumor status. Furthermore, the positive predictive value of the striking findings is 63% and the negative predictive value 100.00% for findings with an exclusion of a local recurrence. The prevalence of local recurrence in the total of examinations is 5%.

When considering uncertain and striking findings combined, there was a sensitivity of 100%, a specificity of 89%, a positive predictive value of 32%, and a negative predictive value of 100%. With a chi-square (*χ*^2^) of 397.97 the correlation is highly significant and the null hypothesis that the findings of the MRI examinations are not related to the actual tumor status must be rejected (Table 5).

A breakdown of the data according to internal and external MRI examinations shows that, amongst internally performed MRIs, there was a prevalence of MRI examinations with a positive tumor status of 4% and a *χ*^2^ of 289.21. Amongst externally performed MRIs, these two values amounted to 18.18% and 74.78. The connection between the radiological findings and the respective tumor status is highly significant for both the internal and external examinations. The sensitivities, the specificities, and the negative predictive values of the internal examinations do not differ from the external ones (Supplementary Tables 1 and 2). However, the prevalence of examinations with a positive tumor status, and the positive predictive value, is higher for the external examinations than for the internal ones.

### 3.4. Cost Benefit Analysis

MRI examinations with no previous event of local recurrence were divided into categories, depending on whether they occurred within the first two years, within the years three to five or after the end of the fifth year after the primary surgery (Figure S1). MRI examinations were again divided into four categories, depending on whether a local recurrence was found in the examination and whether there was a conspicuous previous finding (R1 or R2 resection, or a preceding conspicuous MRI examination). The numbers needed to screen for MRI examinations without a conspicuous previous finding were 30.19 in the first 2 years, 35.85 in the following three years, and 45.29 after five years of follow-up. These values were 5.18, 13, and 5.60, respectively, for cases with suspicious previous findings. Depending on the circumstances of the MRI examination, average costs for discovering a local recurrence therefore range between € 1036 and € 9058 (Table 6). Number needed to screen for the clinical examination between 10.36 and 90.59 was calculated. Multiplication of the number needed to screen results and costs of a single examination resulted in the range between € 1291 and € 11286 for the discovery of local recurrence through clinical examination.

## 4. Discussion

**Figure 3:**
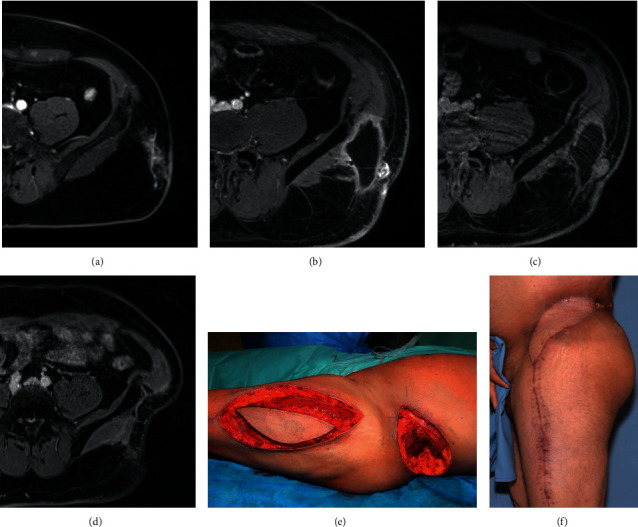
A 42-year-old patient was referred to us after a R1 resection of G2 fibrosarcoma of the left flank at another hospital. The postoperative T1 contrast-enhanced magnetic resonance imaging showed a contrast-enhancing lesion in the subcutaneous tissue (a). An R0 resection was achieved, and adjuvant radiation with 60 Gy followed. The follow-up at six months postoperatively showed a contrast-enhancing nodular lesion, and a sarcoma accumulation (b). The next follow-up after three months demonstrated an increase in size of the nodular lesion (c) so that the patient was recommended to undergo another resection with flap coverage. Histology confirmed the recurrence of the fibrosarcoma. Further course was uneventful, with no suspicion of recurrence after 4 years (d). Resection of the recurrence and coverage with a pedicled anterolateral thigh flap is shown in (e) and the postoperative result in (f).

The goal of local follow-up after sarcoma treatment is the earliest possible discovery of disease recurrence, with the aim of further limb-conserving therapy and reduction of disease-specific morbidity and mortality (Figure 3). Sarcoma are rare and highly heterogeneous tumors, and any research in this field is hampered by small case numbers, as even sarcoma centers see low case numbers of one particular sarcoma type. Accordingly, literature on surveillance for recurrent disease is scarce and unequivocal. Goel et al. reported, in a review of 34 articles, 54 different follow-up strategies [13], and other studies confirm this wide variance between centers [23].

A large number of centers rely on clinical examination alone for local surveillance, reserving imaging for lesions with high risk of recurrence [24–30]. The only prospective study on surveillance for sarcoma found that self-examination of the primary sarcoma site detected the majority of recurrence [31, 32]. Labarre et al. explicitly concluded that MRI is not relevant for detection of local recurrence [33]. At the time, others, including us, rely on MRI for local surveillance [34, 35]. According to the German guidelines on soft tissue sarcoma [5] and American College of Radiology (ACR) Appropriateness Criteria Guidelines [36], MRI is the appropriate modality for surveillance of local recurrence. Several groups found that local surveillance by MRI identified a larger amount of local recurrence than clinical examination [30, 37–39]. Reuther and Mutschler reported than MRI has a higher sensitivity compared to CT in tumors larger than 15 cm [40], and Diana Afonso et al. demonstrated the advantages of using contrast-enhanced MRI [41]. Another group investigated diffusion-weighted MRI, claiming that it is more specific, but less sensitive in fining local recurrence than conventional MRI [42]. Several authors propose adjusting the surveillance strategy according to risk of local recurrence [43–45]. Distinguishing posttreatment changes from recurrent tumor is a major challenge, which necessitates a systematic approach to imaging, with fixed intervals, and adequate reflection of the patient's clinical and surgical history [46]. Fujiki et al. investigated follow-up in patients which received postresection flap coverage of the former tumor site and concluded that MRI is necessary, especially to reveal recurrence occurring under the flap [47].

Aware of the heterogeneity of the literature, and based on the experience that a vast majority of MRI scans have an inconspicuous finding, while posing a significant psychological burden on the patient and a financial strain on the health system, we asked whether the currently recommended frequency of MRI scans is necessary. Our hypothesis was that, as several groups have reported, patients will have palpated the recurrence themselves in many cases before it is imaged by the MRI scan. We hypothesized that this will especially be the case in the distal extremities. The data analysis clearly refuted the hypothesis, i.e., the most cases of recurrence were found by MRI, followed by MRI and examination simultaneously, and then by examination alone. The discrepancy between various studies cannot be accounted for due to the retrospective nature of the studies. The highest-quality data to date comes from an Indian center. The authors performed a prospective noninferiority study, asking whether follow-up intervals can be extended from 3 to 6 months in the first two postoperative years. While the first paper failed to show noninferiority of the 6-month intervals [31], the second publication with a longer follow-up showed that there was no difference in overall survival between the six-month and the three-month groups [32].

The rate of local recurrence in our dataset was somewhat higher compared to the literature, which could be explained by the fact that about 28% of the patients had either metastasized, progressed, or relapsed before the first presentation at our department, which makes further progress and local recurrences more likely [48]. Due to a smaller amount of surrounding tissues, clinical examination and self-examination play a greater role in distal local recurrences than in proximal ones, even though this difference did not reach statistical significance. Clinical symptoms, such as pain, were also more common in distal local recurrences than in proximal ones. However, not only sarcoma recurrence but also biopsies and therapeutic resections can lead to local irritation and pain, some of which assume a chronic course.

Overall, good results were found for sensitivity and specificity, positive and negative predictive value, as well as for the significance of detecting local recurrences by MRI examinations. This is comparable with previous studies, which revealed a sensitivity for MRI scans between 60% and 100% and specificity between 52% and 97.7% [10, 42, 49]. Since there is strong recommendation that sarcoma diagnostics and therapy take place at specialized centers, one could assume that the same holds true for follow-up imaging. Our data suggests that this is not the case, since the MRI examination performed externally had comparable diagnostic quality. This simplifies follow-up logistics for patients who live further away from the university hospital, since they can visit the hospital only once per follow-up term (for clinical examination and discussion of the imaging results), instead of twice (MRI and, a few days apart, examination and discussion). There was a higher prevalence of examinations with a positive tumor status in external MRIs. This could be related to the higher total number of internal examinations and could indicate that internally, due to the better infrastructural and logistical conditions, MRI follow-up was used more generously to clarifying even slightly suspicious findings. In contrast, the patients examined with MRI externally presumably have been preselected more thoroughly.

Although only 8% of the uncertain findings were actually associated with a local recurrence, such findings were followed by further surgical consequence in 34% of the cases. These are the most difficult situations for the patient and the surgeon, and various factors play a role in deciding whether an uncertain MRI examination should be followed by further diagnostic or surgical measures, such as the course of the disease, surgeon's specific knowledge of the surgical site, pathological characteristics (sarcoma type, grading, R-status), and the patient's desire for further diagnosis or therapy.

In the first two years after the initial operation, the MRI examinations became more cost-effective when extending the intervals between these examinations, because the rate of successful local recurrence findings increased. The rising rate of local recurrences at larger intervals between the MRI examinations, however, indicates that those intervals might have been too long. After the first two years, the rate of conspicuous previous findings and the rate of local recurrences decrease. Still, new local recurrences occurred even after more than five years, so that follow-up after more than five years is justified. The number needed to screen of the MRI examinations without any previous findings increases with growing distance from the primary operation, while that of the MRI examinations with previous findings remains at a relatively constant level and even decreases with a very large distance from the primary operation. Even though a clinical examination is cheaper than a MRI examination, the overall cost of finding local recurrence is lower for MRI diagnostics due to better sensitivity and a lower number needed to screen.

## 5. Limitations

Since patients do not receive a biopsy if there is no suspicion of a local recurrence, retrospective statements regarding the actual tumor status are fraught with uncertainty. As described previously, the literature on follow-up after sarcoma is populated by single-center retrospective studies, which often contradict each other. Individual institutions are not likely to change their follow-up protocols, and the ethic committees and patients would understandably be reluctant to agree to randomization with regard to follow-up modality. Furthermore, the heterogeneity of sarcoma and the multiplicity of factors influencing recurrence make direct comparison difficult. To make relevant progress in this field, prospective multicenter trials are a feasible path.

## 6. Conclusions

Our study shows that MRI has an important role in the follow-up after primary treatment of soft tissue sarcomas. In our dataset, MRI diagnostics were superior in detection of local recurrence to the clinical examinations and were thus more cost-effective. However, both methods should be combined because the clinical examination and MRI imaging complement each other. Despite the associated organizational effort and psychological stress, which is exacerbated by the relatively low positive predictive value of MRI diagnostics, we conclude that the MRI intervals should not be extended and advocate continued adherence to the currently valid guidelines.

## Figures and Tables

**Table 1:** Patient demography and tumor characteristics.

		*N* (%)
Total patients		211
Female		104 (49)
Average age in years (±SD)		58.4 ± 17.48
Total sarcomas		216

Location	Epifascial	73 (35)
	Subfascial	118 (56)

Size	T1	72 (34)
	≥ T2	121 (57)

Staging at presentation	M0 N0	167 (79)
	N1 M0	11 (5)
	N0 M1	11 (5)
	N1 M1	2 (1)

R Status after surgery	R0	181 (84)
	R1	24 (11)
	R2	4 (2)

Therapy	Surgery only	138 (65)
	Radiotherapy	73 (35)
	Chemotherapy	20 (9)

**Table 2:** Location, grading, and tumor classification of primary tumors and recurrences.

*N* (%)		Primary tumors	Total local recurrences	Percentage of patients with at least one local recurrence (%)
Head/neck		20 (9)	1 (1)	
Trunk		31 (14)	14 (19)	
Proximal extremity		103 (48)	31 (43)	21
Distal extremity		62 (29)	27 (37)	23

Grading	G1	45 (21)	12 (16)	16
	G2	60 (28)	19 (26)	23
	G3	86 (40)	33 (45)	23
	Unknown	25 (12)	9 (12)	

Liposarcoma		64 (30)	15 (21)	16
Fibroblastic/myofibroblastic sarcomas		45 (21)	19 (26)	29
Undifferentiated/sarcomas		56 (26)	23 (32)	23
Other sarcoma types		51 (24)	16 (22)	24
Smooth muscle sarcoma		13 (6)		
Skeletal muscle sarcoma		2 (1)		
Vascular sarcoma		11 (5)		
Nerve tumors		1		
Extraskeletal osteosarcoma		5 (2)		
Sarcoma of uncertain differentiation		19 (9)		

**Table 3:** Frequency of local recurrence discovery distributed by modality, tumor location, and grading.

*N* (%)		MRI	Clinic	MRI and clinic combined	*p* value
Total		33 (57)	8 (14)	17 (29)	

Location					
Trunk		5 (45)	1 (9)	5 (45)	

Proximal extremity		16 (67)	6 (25)	2 (8)	*p*=0.31 (prox. vs. dist. extremity)
Distal extremity		12 (52)	1 (4)	10 (44)	

Grading	G1	10 (91)	1 (9)	0	*p*=0.04^*∗*^ (G1 vs. G2+G3)
	G2	12 (75)	1 (6)	3 (19)	
	G3	10 (45)	4 (18)	8 (36)	

**Table 4:** Course of further therapy depending on the result of the MRI examination.

*N* (%)	No consequence	Interval shortening	Surgical procedure	Other consequences^*∗*^
Suspected local recurrence	22 (24)	27 (29)	39 (42)	5 (5)
Unable to exclude recurrence	91 (66)	42 (31)	4 (3)	0
Exclusion of a local recurrence	1275 (99)	0	0	1 (0.1%)

^*∗*^Other imaging modalities, e.g., sonography.

**Table 5:** Sensitivity, specificity, and predictive values of MRI diagnostics for discovery of local recurrence. Suspected local recurrence is composed of striking (suspected recurrence) and uncertain (unable to exclude recurrence) findings. These values are explicitly broken down in square brackets; in round brackets are the results of the evaluation using this separation.

	Tumor status: positive	Tumor status: negative	Sum of lines	Predictive values
Suspected local recurrence	68 [58 + 10]	145 [34 + 111]	213 [92 + 121]	Positive: 32% (63%)
Exclusion of local recurrence	0	1186	1186	Negative: 100%
Sum of columns	68	1331	1399	
Sensitivity/specificity	Sensitivity: 100% (85%)	Specificity: 89% (89%)		

**Table 6:** Numbers needed to screen and costs per local recurrence.

Years	Previous conspicuous finding^*∗*^	Number needed to screen	Costs of recurrence detection (€)
0–2	No	30.19	6038.00
	Yes	5.18	1036.00

3–5	No	35.85	7170.00
	Yes	13.00	2600.00

5–10	No	45.29	9058.00
	Yes	5.60	1120.00

^*∗*^Known R1 or R2 resection, and MRI examinations with a conspicuous finding directly preceding the respective examination.

## Data Availability

The data used to support the findings of this study are available from the corresponding author upon request.

## Supplementary Materials

Supplementary Materials Supplementary Figure 1. Relative ratios of MRI examinations within the first two years (a), three to five years (b), and after the end of the fifth year (c) after the primary operation depending on the time interval to the last MRI examination in days; previous findings consist of incomplete resections as well as MRI examinations with a conspicuous finding directly preceding the respective examination. Supplementary Table 1. Sensitivity, specificity, and predictive values of internal MRI diagnostics at the Freiburg University Medical Center for discovery of local recurrence. Suspected local recurrence is composed of striking (suspected recurrence) and uncertain (unable to exclude recurrence) findings. These values are explicitly broken down in square brackets; in round brackets are the results of the evaluation using this separation. Supplementary Table 2. Sensitivity, specificity, and predictive values of external MRI diagnostics (peripheral hospitals and private practice) for discovery of local recurrence. Suspected local recurrence is composed of striking (suspected recurrence) and uncertain (unable to exclude recurrence) findings. These values are explicitly broken down in square brackets; in round brackets are the results of the evaluation using this separation.
