# Supplementary material for: Efficacy and Cost-Benefit Analysis of Magnetic Resonance Imaging in the Follow-Up of Soft Tissue Sarcomas of the Extremities and Trunk
Source: J Oncol. 2021 Apr 27;2021:5580431. doi: 10.1155/2021/5580431 (PMC8100420; doi:10.1155/2021/5580431)
Supplement: Supplementary Materials — Supplementary Figure 1. Relative ratios of MRI examinations within the first two years (a), three to five years (b), and after the end of the fifth year (c) after the primary operation depending on the time interval to the last MRI examination in days; previous findings consist of incomplete resections as well as MRI examinations with a conspicuous finding directly preceding the respective examination. Supplementary Table 1. Sensitivity, specificity, and predictive values of internal MRI diagnostics at the Freiburg University Medical Center for discovery of local recurrence. Suspected local recurrence is composed of striking (suspected recurrence) and uncertain (unable to exclude recurrence) findings. These values are explicitly broken down in square brackets; in round brackets are the results of the evaluation using this separation. Supplementary Table 2. Sensitivity, specificity, and predictive values of external MRI diagnostics (peripheral hospitals and private practice) for discovery of local recurrence. Suspected local recurrence is composed of striking (suspected recurrence) and uncertain (unable to exclude recurrence) findings. These values are explicitly broken down in square brackets; in round brackets are the results of the evaluation using this separation. [file 5580431.f1.zip › 5580431.f1/Supp.Table1.docx]

**Supplementary table 1.** Sensitivity, specificity, and predictive values of internal MRI diagnostics at the Freiburg university medical center for discovery of local recurrence. Suspected local recurrence is composed of striking (suspected recurrence) and uncertain (unable to exclude recurrence) findings. These values are explicitly broken down in square brackets; in round brackets are the results of the evaluation using this separation.

|  | **Tumor status: positive** | **Tumor status: negative** | **Sum of lines** | **Predictive values** |
| --- | --- | --- | --- | --- |
| **Suspected local recurrence** | 46  [39 + 7] | 135  [33 + 102] | 181  [72 + 109] | positive:  25%  (54%) |
| **Exclusion of local recurrence** | 0 | 1097 | 1097 | negative:  100%  (100%) |
| **Sum of columns** | 46 | 1232 | 1278 |  |
| **Sensitivity/specificity** | sensitivity:  100%  (85%) | specificity:  89%  (89%) |  |  |
